# Abnormal Functional Connectivity Between Cerebral Hemispheres in Patients With High Myopia: A Resting FMRI Study Based on Voxel-Mirrored Homotopic Connectivity

**DOI:** 10.3389/fnhum.2022.910846

**Published:** 2022-06-23

**Authors:** Yi Cheng, Xiao-Lin Chen, Ling Shi, Si-Yu Li, Hui Huang, Pei-Pei Zhong, Xiao-Rong Wu

**Affiliations:** Department of Ophthalmology, The First Affiliated Hospital of Nanchang University, Nanchang, China

**Keywords:** high myopia, voxel-mirrored homotopic connectivity, functional magnetic resonance imaging, putamen, fusiform gyrus

## Abstract

**Purpose:**

To study the changes in functional connections between the left and right hemispheres of patients with high myopia (HM) and healthy controls (HCs) by resting functional magnetic resonance imaging (fMRI) based on voxel-mirrored homotopic connectivity (VMHC). To study the changes in resting-state functional connectivity (rsFC) between the left and right hemispheres of patients with HM and healthy controls (HCS) at rest by using resting functional magnetic resonance imaging (fMRI) based on voxel-mirror homotopy connectivity (VMHC).

**Patients and Methods:**

A total of 89 patients with HM (41 men and 48 women) and 59 HCs (24 men and 35 women) were collected and matched according to gender, age, and education level. The VMHC method was used to evaluate the changes in rsFC between cerebral hemispheres, and a correlation analysis was carried out to understand the differences in brain functional activities between the patients with HM and the HCs.

**Results:**

Compared with the HCs, the VMHC values of the putamen and fusiform in the HM group were significantly lower (voxel-level *p* < 0.01, Gaussian random field correction cluster level *p* < 0.05).

**Conclusion:**

This study preliminarily confirmed the destruction of interhemispheric functional connection in some brain regions of the patients with HM and provided effective information for clarifying the neural mechanism of patients with HM.

## Introduction

High myopia (HM) is a worldwide health problem that has attracted widespread attention. It seriously affects the quality of life of patients. The rate of incidence is increasing year by year. It affects about 2.9% of the global population (Holden et al., 2015). HM refers to myopia with diopter > −6D, mostly axial myopia, which is characterized by pathological and degenerative changes in the retina, choroid, and sclera, resulting in visual impairment. Therefore, the harm is great (Hsu et al., 2014). Eyeground structures such as the retina and optic nerve are closely related to the brain, especially visual-related areas (Wang et al., 2020). One study has shown that the retinal nerve fiber layer of patients with HM becomes thinner and that the optic papilla is deformed (Qu et al., 2018). Zhai et al. (2016) found that the visual function of patients with HM was abnormal, the functional connection of the posterior cingulate cortex/precuneus decreased, and the function of other brain regions changed. Therefore, they believed that abnormal visual experiences would lead to abnormal brain structure and function. In addition, a correlation analysis based on functional connectivity density (FCD) mapping and seeding showed that HM can lead to changes in brain morphology and functional connectivity density (Li et al., 2012; Zhai et al., 2016). Consequently, HM may lead to changes in brain functional activities.

In order to evaluate the changes in brain activity, functional magnetic resonance imaging (fMRI) is increasingly conducted in studies on HM. MRI has the characteristics of multi-sequence, multi parameters, no ionizing radiation, routine imaging, and functional imaging. It can find changes in brain activity and function in patients with HM, and it has a great application value. A recent study used functional magnetic resonance imaging to evaluate the activity of occipital visual cortex in patients with lens induced high myopia (IHM) under different visual stimulation. The results showed that the activity of visual cortex in patients with IHM was significantly lower than that in the normal group (Mirzajani et al., 2017). Patients with HM have certain changes in visual pathway area and limbic system structure (Huang et al., 2018). Homotopic functional connectivity is the connection between mirror regions of the cerebral hemisphere, and it is also one of the most remarkable features of the internal functional structure of the brain (Salvador et al., 2005; Stark et al., 2008). It may reflect that inter-hemispheric communication plays a very important role in integrating brain function based on coherent cognition and behavior. At the same time, it has significant spatial variability related to function and is disturbed by a variety of pathological conditions (Mancuso et al., 2019). Previous studies related to MRI have found that HM affects brain neural activity and functional connection, but the specific mechanism has not been fully clarified.

Voxel-mirrored homotopic connectivity (VMHC) is a method proposed by Stark DE (Stark et al., 2008) to explore the differences in functional connections between voxels in bilateral hemispheric symmetrical systems and evaluate their coordination. It is a functional magnetic resonance imaging technology used to explore brain tissue patterns based on resting functional connections. This method needs to conduct anatomical structure correction to make the bilateral cerebral hemispheres basically symmetrical and calculate the time-series correlation between each voxel and its contralateral hemisphere allele. The lower the VMHC value, the worse the coordination of allelic voxel functional activities between the bilateral cerebral hemispheres. On the contrary, the higher VMHC value, the coordination will be better. Different MRI analysis methods have different emphases in expressing the functional changes of brain regions. VMHC not only directly compares functional connections between cerebral hemispheres in the resting state but also accurately and effectively evaluates changes in functional connections between cerebral hemispheres related to patients' behavior and cognition. It has certain efficiency, stability, and safety in studies on brain information integration. In recent years, more and more studies have focused on VMHC and found that it is related to a variety of diseases and functional states, and has a good correlation. Kelly et al. found that there is an association between long-term exposure to cocaine and large-scale brain circuit interruption supporting cognitive control through the VMHC method (Kelly et al., 2011). Luo et al. (2018) found that the VMHC value was lower in some brain regions of multi-domain amnestic mild cognitive impairment patients and showed more severe interhemispheric communication defects than that of single-domain amnestic mild cognitive impairment patients. In addition, there were VMHC abnormalities in multiple brain regions of patients with type 2 diabetes, indicating that the functional coordination between the same brain regions was generally impaired (Zhang Y. et al., 2021). In conclusion, rs-fMRI analysis based on VMHC can provide additional information beyond the classical FC index for understanding the neural mechanism of executive function changes between cerebral hemispheres, and VMHC is a reliable neural index for brain function reorganization. Through previous studies, it has also been found that VMHC has been used for the analysis of a variety of eye diseases, such as primary open-angle glaucoma (Liu et al., 2008), monocular blindness (Shao et al., 2018), and strabismus amblyopia (Zhang S. et al., 2021). We speculate that patients with HM, which affects brain visual imaging, may have abnormal brain functional activities. Therefore, this study discusses changes in cerebral hemisphere functional connection in patients with HM by VMHC analysis, to provide a new idea for the neural mechanism of HM.

## Materials and Methods

### Participants

Participants were selected from the hospital during the period from September 2018 to September 2020. The cohort included 89 patients with HM and 59 HCs without ametropia. The inclusion criteria for patients with myopia were as follows: (1) aged 18–60 years, (2) meeting the diagnostic criteria of high myopia, (3) right-hand dominance, and (4) no other ophthalmic diseases. The exclusion criteria were: (1) other ophthalmic diseases, (2) unilateral myopia, (3) abnormal brain structure such as tumor or subdural hematoma, (4) mental diseases and unable to cooperate, and (5) complications of HM including HM optic neuropathy and HM macular degeneration. The age, sex, hand advantage, and education level of the HCs were matched with those of the HM group and met the following criteria: (1) no ametropia and other ophthalmic diseases, (2) no mental diseases, and (3) normal routine brain magnetic resonance imaging. All the subjects underwent MRI scanning, and those with incomplete MRI data or excessive head movements were excluded. The study was approved by the medical ethics committee of the First Affiliated Hospital of Nanchang University, and all the participants were informed and agreed.

### MRI Acquisition

All MRI data were collected with a Siemens Trio 3.0T scanner by implementing an 8-channel head coil. MRI scanning was performed on each subject. Whole-brain T1-weighted images were obtained with magnetization-prepared gradient echo image (MPRAGE) with these parameters: repetition time = 1,900 ms, echo time (TE) = 2.26 ms, thickness = 1, no intersection gap, acquisition matrix =2 56 × 256, field of view (FOV) = 240 × 240 mm2, and flip angle = 12°. Functional images were obtained using a gradient-echo-planar imaging sequence.

### Data Preprocessing in Functional Magnetic Resonance Imaging

The preprocessing reason brain imaging data processing and analysis toolbox (DPABI, http://rfmri.org/dpabi) are completed based on statistical parameter mapping (SPM12), which runs on MATLAB 8.4.0 (MathWorks, Natick, MA, United States). The main preprocessing steps were: discard the first 10 volumes, slice timing, spatial rearrangement, head motion correction, individual registration between high-resolution T1 and EPI images, spatial normalization, and spatial smoothing that can reduce the registration error and increase the normality of statistical data. The RS fMRI data set is registered in the space of Montreal Neurological Institute (MNI) and resampled to a 3 × 3 × 3 mm^3^ cube voxel; head movement data with a maximum translation of more than 2 mm or a maximum rotation of 2° were excluded from the final analysis. The preprocessed data are divided into typical frequency bands (0.01–0.1 Hz). Linear regression is performed on covariates such as head movement, whole-brain parenchyma, and cerebrospinal fluid signals to reduce the impact (including white matter, cerebrospinal fluid, and head movement parameters based on Friston 24 parameter model) (Friston et al., 1996).

### Statistical Analysis of VMHC

The value of VMHC is calculated as the Pearson correlation coefficient between the voxel time series between each pair of mirror hemispheres (Zuo et al., 2010). The REST software was used to calculate the Pearson's correlation between each group of symmetrical alleles in both cerebral hemispheres one by one, and then the fisher-z transform was performed on these correlation values. The results of correlation values formed the VMHC diagram and were used for subsequent group-level analysis. To reduce the difference between different subjects, it is also necessary to standardize the mean of the whole brain VMHC map.

### Statistical Analysis

This study was tested with the SPSS software (version 13.0; SPSS Inc., Chicago, IL, United States). The changes in VMHC z-diagram between the healthy control group and the HM group were evaluated by two-sample *t*-tests. All important clusters were reported on MNI coordinates, and the T value of peak voxels was determined (voxel-level *P* < 0.01, Gaussian random field [GRF] correction, cluster-level *P* < 0.05).

## Results

### Basic Information

The basic information of patients with HM and the healthy control group is shown in Table 1.

**Table 1:** General characteristics of the patients.

	**Age(years)**	**Female/Male**	**ALM(OD)**	**ALM(OS)**
HM	26.235 ± 0.462	48/41	26.670 ± 0.874	26.580 ± 0.985
HCs	25.783 ± 0.102	35/24	23.900 ± 0.971	23.740 ± 0.693

*ALM, axial length; HM, high myopia; HCs, healthy controls; OD, oculus dexter; OS, oculus sinister*.

### VMHC Group Differences

**Figure 1:**
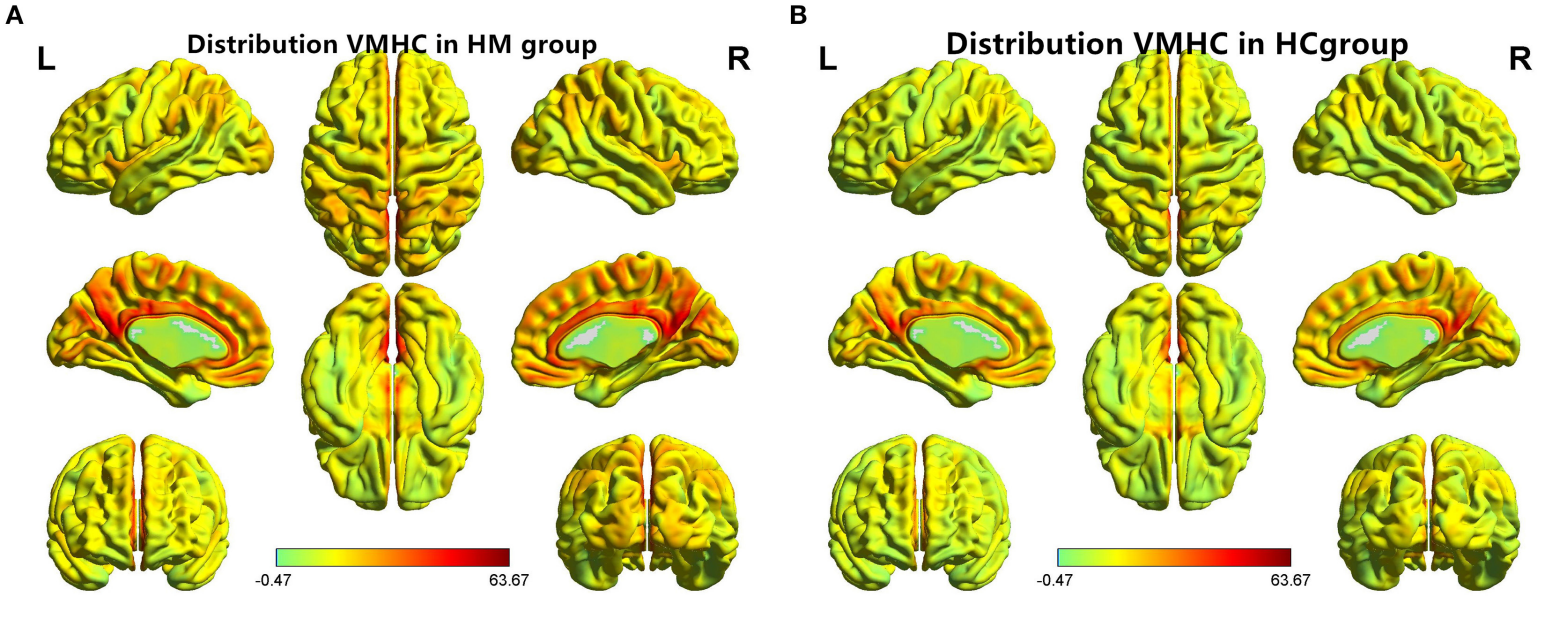
Distribution patterns of VMHC were observed at the group level in patients with **(A)** HM and **(B)** HCs. VMHC, voxel-mirrored homotopic connectivity; HM, high myopia; HCs, healthy controls.

**Figure 2:**
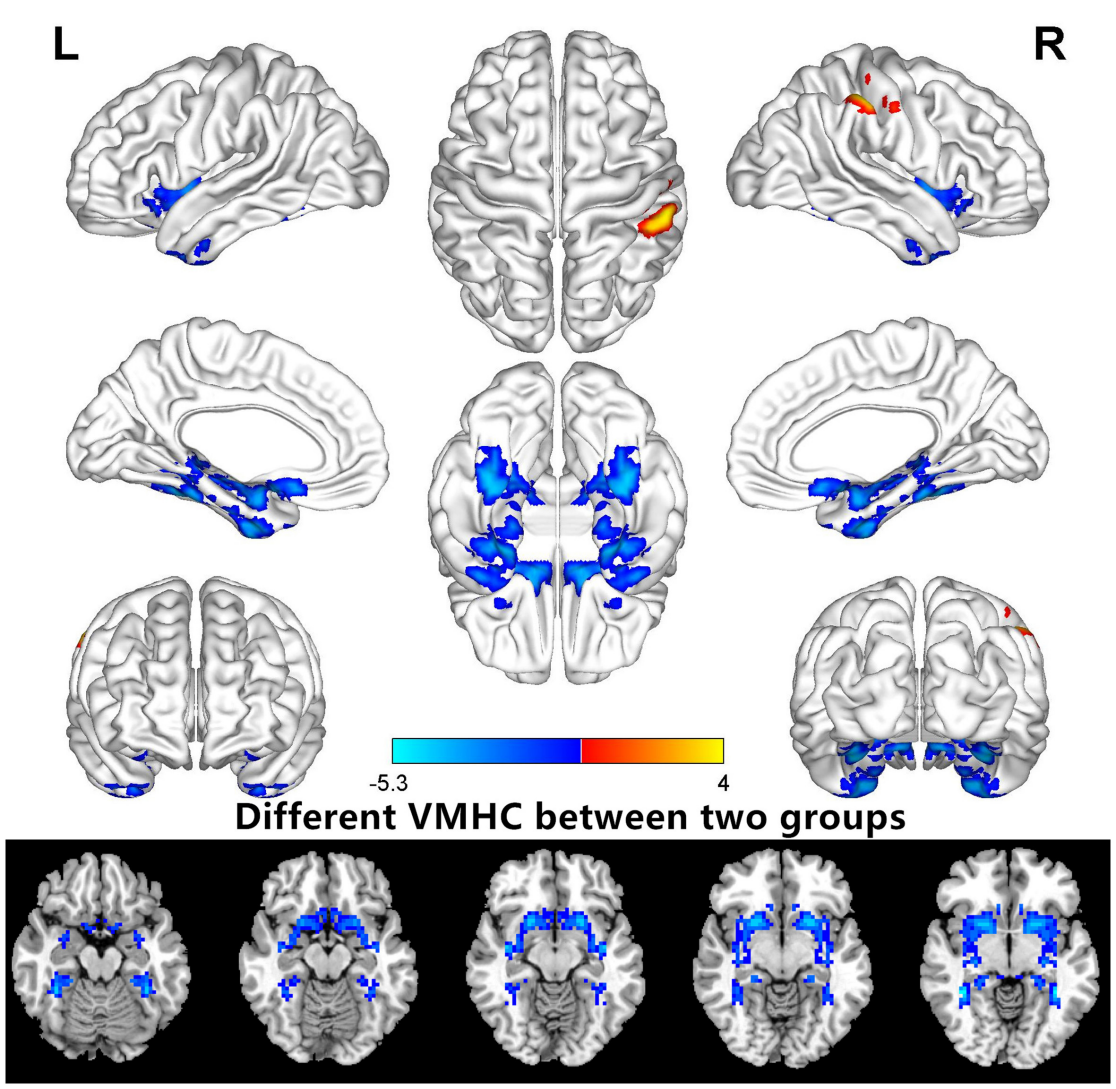
Different VMHCs between the HM and HC groups (voxel level *P* < 0.01, GRF correction, cluster level *P* < 0.05). VMHC, voxel-mirrored homotopic connectivity.

VMHC values changed between the HM group and the HC group (Figure 1); Compared with HCS, the VMHC in the lenticular nucleus and fusiform gyrus decreased in patients with HM (Figure 2, Table 2).

**Table 2:** Different voxel-mirrored homotopic connectivity (VMHC) values of brain areas between the HM and HC groups (voxel level *P* < 0.01, Gaussian random field, GRF, correction).

**Conditions**	**L/R**	**Brain regions**	**MIN coordinates**			**Cluster size**	***T*-value**
			**X**	**Y**	**Z**		
HM < HC							
1		Putamen	21	12	±9	664	−4.8082
2		Fusiform	±36	−39	−21	210	−5.2993

*T: there were significant differences in the statistical values of peak voxels between the two groups. X, Y, and Z are the coordinates of the main peak position in the Montreal Neurological Institute (MNI) space (voxel level P < 0.01, GRF correction). HM, high myopia; HCs, healthy controls*.

## Discussion

In this study, we evaluated the VMHC difference between the HM group and the HC group. The study found that compared with the HCs, the VMHC values of the putamen and fusiform gyrus of the patients with HM were decreased.

The putamen is a structure located under the cortex and a part of the dorsal striatum of the basal ganglia. It is considered to be related to reinforcement learning, motor control, and language function (Vinas-Guasch and Wu, 2017). Recently, it has been found that the putamen is closely related to processes that visually guided and internally guided force control in humans. A study on changes in visual movement and functional activities in brain regions found that 91% of caudate cells and 65% of putamen cells changed when monkeys performed visual tasks. Therefore, the activity of neurons in the caudate and putamen during a visuomotor task will be activated whether there is motor behavior or not (Romero et al., 2008). Li et al. found that the regional homology (ReHo) of bilateral putamen in patients with idiopathic rapid eye movement sleep behavior disorder (iRBD) was significantly reduced, the tracer uptake of bilateral putamen and left caudate nucleus was also significantly reduced, and abnormal spontaneous brain activity of bilateral putamen in patients with iRBD was detected when studying the correlation between iRBD and the putamen (Li et al., 2019). It can be seen from the above that the change in the putamen is related to the abnormal state of the body. When studying changes in brain structure between patients with HM and HCs, Huang et al. (2018) found that the global gray matter volume (GMV) of the bilateral putamen in patients with HM was increased. The authors speculated that HM may lead to structural changes in the bilateral putamen, to compensate for the motor function of HM. In our study, we found that compared with the HC group, the VMHC value of the putamen in patients with HM was decreased, which may be related to abnormal changes in this area due to the abnormal visual experience and behavior of HM. It is consistent with the above research results. Hence, we speculate that HM will lead to disorder in the putamen connection and affect visual function.

The fusiform gyrus is located in the middle and bottom of the visual joint cortex. It is the largest macro-anatomical structure in the advanced visual cortex and has a wide range of functions; however, it is highly controversial (Cohen et al., 2000; Huth et al., 2012; Cukur et al., 2013). At first, it was well-known for its facial-recognition function, but with the deepening of research, it was found that it was more involved in processing high-order visual information, especially related to face, body, and stimuli characterized by high spatial frequency, and was responsible for the recognition of secondary classification of objects (Weiner and Zilles, 2016; Rokem et al., 2017). Moreover, the fusiform gyrus has a special visual processing mechanism for text and objects (Chen et al., 2019). Tanja Kassuba et.al. found that the lateral occipital cortex (LO), fusiform gyrus, and intraparietal sulcus (IPS) play an important role in the integration of visual-tactile objects (Kassuba et al., 2014). In addition, a study on the frequency-dependent spontaneous neural activity of primary angle-closure glaucoma found that the ALFF values of multiple brain regions were changed. The ALFF values in the bilateral frontal lobe, right fusiform gyrus, and right posterior cerebellar lobe were higher in slow band 5 than in slow band 4. It was considered that the abnormal spontaneous neural activity of patients with PACG indicates that the cognitive, visual, and emotional functions of these patients are impaired (Jiang et al., 2019). In our study, we found that the VMHC value of the fusiform gyrus in the patients with HM was decreased. We speculated that abnormal visual experience may lead to structural changes in the fusiform gyrus and even affect its functions, such as visual and recognition functions.

However, there are some limitations to this experiment. For example, the sample size is not large enough, and the head movement and cerebrospinal fluid movement effects of the patients during examination have a certain impact on the experiment. However, to minimize such effects, we have strictly selected qualified examination results and eliminated the effects of movement on the brain through statistical methods. In addition, the results of this study were obtained in the resting state of participants. In future studies, we will also combine the resting-state and task-state fMRI to research the changes of abnormal brain regions in different states. At the same time, we will expand the sample size and improve the applicability and accuracy of the results.

## Conclusion

In conclusion, compared with the HCs, VMHC values in different brain regions of the patients with HM have different changes, suggesting that HM may cause changes in functional connections between cerebral hemispheres in some regions, resulting in functional damage, which may become a breakthrough in studying the divine mechanism of changes in visual and motor functions in HM.

## Data Availability Statement

The raw data supporting the conclusions of this article will be made available by the authors, without undue reservation.

## Ethics Statement

The studies involving human participants were reviewed and approved by The First Affiliated Hospital of Nanchang University. The patients/participants provided their written informed consent to participate in this study.

## Author Contributions

X-RW: guidance on the content of this article. X-LC: charts and diagrams graphic typesetting. S-YL, LS, HH, and P-PZ: clinical data collection. All authors contributed to the article and approved the submitted version.

## Funding

This work was supported by Key research plan of Jiangxi Provincial Department of Science and Technology (No. 20192BBG70042), Jiangxi Province Education Department Key Foundation (No. GJJ160033), Health Development Planning Commission Science Foundation of Jiangxi Province (No. 20185118), Technology and Science Foundation of Jiangxi Province (No. 20141BBG70027), Jiangxi Province Education Department Scientific Research Foundation (No. GJJ13147), Health and Family Planning Commission Traditional Chinese Medicine Foundation of Jiangxi Province (No. 2017A001), and Basic Health Appropriate Technology Spark Promotion Program of Jiangxi Province (No. 20188007).

## Conflict of Interest

The authors declare that the research was conducted in the absence of any commercial or financial relationships that could be construed as a potential conflict of interest.

## Publisher's Note

All claims expressed in this article are solely those of the authors and do not necessarily represent those of their affiliated organizations, or those of the publisher, the editors and the reviewers. Any product that may be evaluated in this article, or claim that may be made by its manufacturer, is not guaranteed or endorsed by the publisher.
